# Dissemination of Information to Foreigners in Preparation for Natural Disasters

**DOI:** 10.31662/jmaj.2024-0327

**Published:** 2025-01-31

**Authors:** Hatsune Kido, Soichiro Saeki

**Affiliations:** 1 Department of Medical Education, Kurashiki Central Hospital, Okayama, Japan; 2 Emergency Medicine and Critical Care, Center Hospital of the National Center for Global Health and Medicine, Tokyo, Japan; 3 Division of Public Health, Department of Social Medicine, Graduate School of Medicine, Osaka University, Osaka, Japan

**Keywords:** Plain Japanese, Easy Japanese, Natural Disasters, Disaster Planning, Earthquakes, Emigrants and Immigrants, Japan

Earthquakes are frequent in Japan, underscoring the critical importance of earthquake preparedness for all, including foreign residents. Significant seismic events, such as the January 1, 2024 earthquake in the Noto Peninsula, highlight the destructive potential of such disasters. Therefore, the findings of Koike et al. ^(1)^ on foreign residents’ preparedness for major earthquakes deserves serious attention. Additional considerations are proposed to further enhance safety during such events.

First, promoting “plain Japanese” in disaster communications should be prioritized. Designed to be easily understood by non-native speakers, plain Japanese can assist foreign residents in emergencies ^(2)^. Even those with intermediate to advanced Japanese proficiency (N1 to N3) may experience difficulty processing critical information during a crisis caused by panic. Ensuring that essential disaster information is disseminated in plain Japanese would facilitate more effective communication and response during emergencies.

Second, disaster-related information must be made accessible to all foreign residents, particularly those from Southeast Asia, where technical interns are increasingly prevalent. Many websites offer information in major languages, such as English, Chinese, and Korean; however, the coverage of other languages remains limited. One possible solution is machine translation, as implemented by the Tokyo Metropolitan Government ^(3)^. Multilingual disaster communication will help ensure that all residents, regardless of language barriers, can respond appropriately during emergencies.

Third, in disaster situations, individuals may struggle to communicate in Japanese because of confusion. To address this need, preparing printouts or pointing boards with critical phrases such as “I need water” or “I feel unwell” in Japanese and the individual’s native language could help convey basic needs (Figure 1). Additionally, medical professionals must prepare to assist non-Japanese speakers ^(4)^, making the role of medical interpreters crucial in such situations. Establishing systems for interpreters during routine times would ensure smoother medical care during disasters. Although plain Japanese is used in disaster response, it has yet to be fully adopted in medical settings ^(2), (5)^, presenting potential challenges that must be addressed.

**Figure 1.**
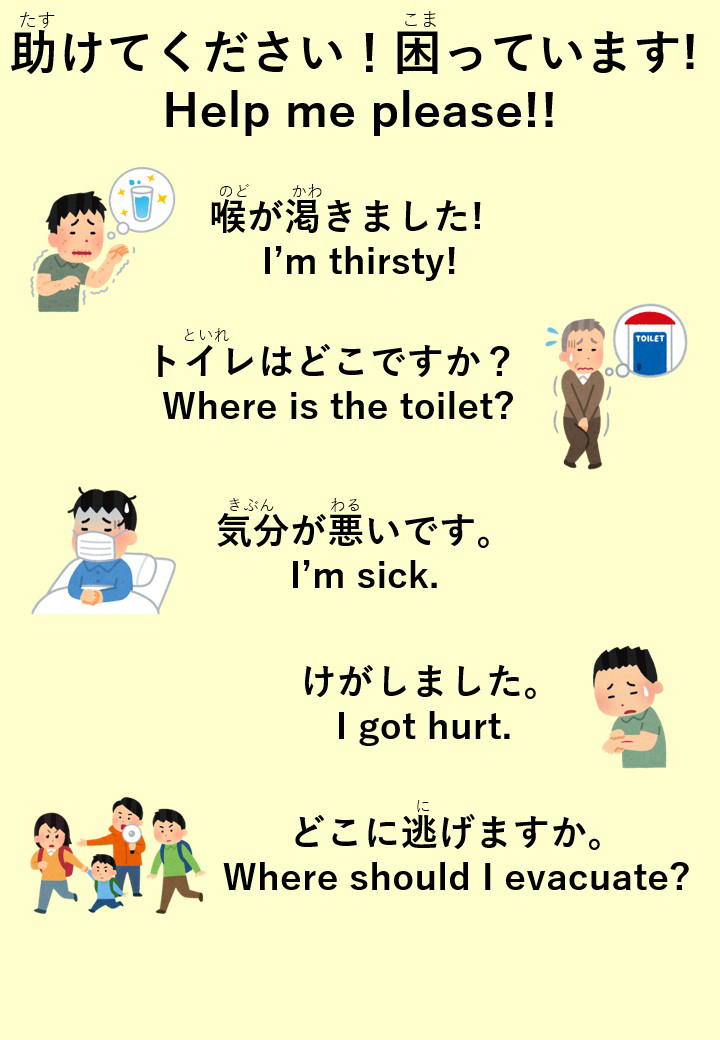
Pointing Board for Foreigners During Natural Disasters An example of a pointing board in English (prepared by the authors).

Japan has made considerable advancements in adapting to natural disasters; however, additional efforts are necessary to develop a more holistic and comprehensive disaster preparedness approach. Simulating real disaster scenarios during prevention planning is imperative to ensure that preparedness strategies are practical and effective. As Japanese society becomes increasingly diverse, future disaster management must consider the safety and well-being of foreign residents.

## Article Information

### Conflicts of Interest

None

### Acknowledgement

The authors thank our colleagues for helpful discussions on this topic. The authors acknowledge the use of Grammarly (Grammarly Inc, San Fransico, USA) for primary language editing. The views expressed in this manuscript are those of the authors and do not necessarily represent the authors’ institutions.

### Author Contributions

Both authors contributed to creating the synopsis of the manuscript. HK prepared the first draft of the manuscript. SS critically reviewed the manuscript. Both authors read and approved the final version of the manuscript. Artificial intelligence technology was used for the language editing process, and such content was reviewed by the author. The author’s institution played no role in the conceptualization of this manuscript.

### Approval by Institutional Review Board (IRB)

Not applicable.
